# Partial and full root-zone drought stresses account for differentiate root-sourced signal and yield formation in primitive wheat

**DOI:** 10.1186/s13007-019-0461-5

**Published:** 2019-07-12

**Authors:** Asfa Batool, Zheng-Guo Cheng, Nudrat Aisha Akram, Guang-Chao Lv, Jun-Lan Xiong, Ying Zhu, Muhammad Ashraf, You-Cai Xiong

**Affiliations:** 1 State Key Laboratory of Grassland Agro-Ecosystems, School of Life Sciences, Lanzhou University, Lanzhou, 730000 China; 2 Department of Botany, GC University, Faisalabad, 38040 Pakistan; 3 Faculty of Agriculture, The University of Sargodha, Sargodha, 40100 Pakistan

**Keywords:** Abscisic acid, Drought, Partial root-zone stress, Yield formation, Water use efficiency

## Abstract

**Background:**

Partial and full root-zone drought stresses are two widely used methods to induce soil drying in plant container-culture experiments. Two methods might lead to different observational results in plant water relation, such as non-hydraulic root-sourced signal (nHRS). We compared partial and full stress methods to induce nHRS in two diploids (MO1 and MO4) and two tetraploids (DM 22 and DM 31) wheat varieties under pot-culture conditions. Partial root-zone stress (PS) was performed using split-root alternative water supply method (one half wetting and the other drying) to induce the continuous operation of nHRS, and full root-zone stress (FS) was exposed to whole soil block to induce periodic operation of nHRS since jointing stage.

**Results:**

We tested the two drought methods whether it influenced the nHRS mediated signalling and yield formation in primitive wheat species. Results showed that partial root-zone stress caused more increase in abscisic acid (ABA) production and decline in stomatal closure than full root-zone stress method. The incline in ABA was closely related to triggering reactive oxygen species (ROS) generation, and reducing cytokinin synthesis which, thereby, led to crosstalk with other signalling molecules. Furthermore, PS up-regulated the antioxidant defense system and proline content. Water use efficiency and harvest index was significantly increased in PS, suggesting that PS was more likely to simulate the occurrence of nHRS by increasing the adaptive strategies of plants and closer to natural status of soil drying than FS.

**Conclusion:**

These findings lead us to conclude that partial root-zone stress method is more feasible method to induce nHRS which has great capacity to reduce water consumption and enhance plant adaptation to constantly changing environment. These observations also suggest that different root-zone planting methods can be considered to improve the plant phenotypic plasticity and tolerance in water-limited rainfed environments.

**Electronic supplementary material:**

The online version of this article (10.1186/s13007-019-0461-5) contains supplementary material, which is available to authorized users.

## Background

Plant root system can sense drying soil and send chemical signals to above-ground parts, closing the stomata and maintaining leaf water status [1, 2]. This phenomenon is involved in a series of eco-physiological and biochemical mechanisms to cope with drought stress in higher plants [3, 4]. According to root-to-shoot communication theory, root system can produce phytohormones such as abscisic acid (ABA) and cytokinins (CKs), i.e. non-hydraulic root-sourced signals (nHRS), and transfer them to the leaves, thereby inducing stomatal closure before leaf water status significantly decreases [5, 6]. During this process, leaf and other major organs can maintain osmotic adjustment, and improve antioxidant defense, resulting drought tolerance [3, 7]. This early-warning response is activated at different levels from cell, tissue, organ to whole plant [8]. In most cases, physiological characteristics and functional roles of nHRS vary from plant species and genotypes [9].

Plant hormones such as ABA and cytokinin (CK) produce in response to drying soil, however they play differentiate roles [10] in adaptation to drought conditions. CK might act as one of major root-sourced signals to impel stomatal opening and work antagonistically against ABA [11]. However, this hypothesis is not supported by more experimental observations. Actually, exogenous CK application help improve drought tolerance, mainly because CK can follow a cooperative mechanism to work with ABA. In addition, CK application can help improve photosynthetic rate and water use efficiency through activating antioxidant defense system in many plant species [12, 13]. Under abiotic stresses, CK can modulate the activities of antioxidant enzymes such as catalase (CAT), peroxidase (POD) and superoxide dismutase (SOD) in the leaves [14, 15]. Therefore, it can maintain plant metabolism and prevent major organs and tissues from stress-induced oxidative damage [16]. Exogenous ABA application on leaves can enhance grain yield in field-grown wheat in dry growing seasons [17]. On the other hand, there exists a positive correlation between leaf ABA biosynthesis and stomatal closure in response to drying soil [18]. Early triggering of stomatal closure is frequently associated with enhanced antioxidant defense response and help increase grain yield and WUE in wheat crop [19]. However, it is so far unclear how the interaction between ABA and CK affects yield formation and water use, and how its physiological mechanism is.

Over last decades, there were two major methods to be employed to investigate the physiological and agronomic characteristics of nHRS in maize and wheat, including root-splitting experiment [1, 20, 21] and full root-zone drying experiment [9, 19, 22]. Yet, the two methods led to different results due to the difference in water supply strategies [23]. The comparison on two methodologies may provide a wider insight into the functional role of nHRS in dryland crops [24, 25]. Among these attempts, nHRS is generally found to induce the enhancement of protective defense response when nHRS operates. Protective defense system is mainly featured by the production of reactive oxygen species (ROS) and then increase in antioxidant defense system to scavenge the ROS [26]. Under mild and intermediate drought stress, the oxidative damage to lipid membrane is a major physiological phenomenon [27]. Also, low concentrations of ROS such as hydrogen peroxide (H_2_O_2_), hydroxyl radicals (·OH) and superoxide anion radicals (O_2_^−^) are generally viewed as critical adaptive strategies. During the operation of nHRS, an antioxidant defense system might be activated by chemical signal substance ABA [28]. However, the relevant adaptive strategies mostly vary from crop genotypes [29] and very few studies have addressed this issue in primitive wheat.

Based on the number of chromosomes, wheat is usually classified into three groups: diploid (2x = 2n = 14, where n = 7), tetraploid (2x = 4n = 28), and hexaploid (2x = 6n = 42). Domesticated wheats are widely found at all three ploidy levels, whereas primitive wheats only exist at diploid and tetraploid levels [30]. From the perspective of evolution theory, primitive wheat may preserve a series of adaptive strategies under water limiting condition and maintain the reproductive capability [31, 32]. As is well known, primitive wheat species are the genetic donors of modern wheat germplasm resource, with critical unknown merit strategies to adapt to dry environment in their genome. The unique strategies may change the pattern of biomass allocation in diploid and tetraploid wheats [33, 34].

Wheat species differing in ploidy frequently vary in their abilities to simulate the occurrence of nHRS and modulate the induction of soil drying in response to water deficit conditions [21]. However, the comparison of biochemical and growth responses between two soil drying methods, partial and full root-zone, with the induction of non-hydraulic root signal is not well documented. Therefore, the specific objectives of present study include (1) comparing the role of two different root-zone drying methods to simulate the occurrence of nHRS and the induction of soil drying, (2) verifying an improved method to estimate the nHRS mediated signalling and their crosstalk with other biochemical and physiological signals; and (3) to explore the potential of partial root-zone drying method on full root-zone drying to quantify root-sourced signal and yield formation in primitive wheat.

## Results

### Non-hydraulic root-sourced signal (nHRS) and the changes in leaf ABA and ZR concentrations

The nHRS is generally judged as there is a significant decrease in stomatal conductance status without detectable change in leaf water status in the plant, exposed to drying soil. In this study, leaf relative water content (RWC) was used to express water status. As shown in Table 1, across all the species, leaf RWC did not change significantly while stomatal conductance decreased significantly in both drought treatments (PS and FS) (Table 1) and was lowest under PS in four wheat varieties. Regardless of wheat varieties, the photosynthetic rate was substantially affected by nHRS. In general, the photosynthetic rate was decreased significantly while there was no significant difference between PS and FS (Table 1).

**Table 1:** Leaf relative water content (RWC, %), stomatal conductance (*gs*), photosynthetic rate (*Pn*), and transpiration rate (*E*) at the flowering stage, and total water consumption, water use efficiency for grain yield (WUE_G_), and water use efficiency for aboveground biomass (WUE_AGB_) at the maturity stage of four wheat varieties having different ploidy level subjected to three drought treatments (WW, FS and PS)

Species	Varieties	Treatments	Leaf relative water content (%)	Stomatal conductance (mmol H_2_O m^−2^ s^−1^)	Photosynthetic rate (µmol CO_2_ m^−2^ s^−1^)	Transpiration rate (mmol H_2_O m^−2^ s^−1^)	Total water consumption (L/plant)	WUE_G_	WUE_AGB_
Diploid	MO1	WW	86.5 ± 2.0a	73.86 ± 3.3c	8.12 ± 0.4b	4.73 ± 0.4b	1.83 ± 0.05c	1.14 ± 0.04b	3.01 ± 0.10b
		FS	82.0 ± 4.0a	47.41 ± 5.0b	5.49 ± 0.6a	2.93 ± 0.1a	1.34 ± 0.02b	1.04 ± 0.05a	2.55 ± 0.02a
		PS	81.3 ± 2.0a	35.01 ± 2.0a	6.26 ± 0.2a	2.74 ± 0. 3a	1.12 ± 0.06a	0.99 ± 0.04a	2.72 ± 0.19ab
	MO4	WW	76.1 ± 3.0a	86.11 ± 5.2c	10.01 ± 0.4b	4.47 ± 0.3b	1.66 ± 0.05c	1.18 ± 0.02b	3.10 ± 0.13b
		FS	74.7 ± 2.0a	45.30 ± 4.5b	6.97 ± 0.2a	2.43 ± 0.3a	1.15 ± 0.03b	0.98 ± 0.04a	2.54 ± 0.05a
		PS	73.5 ± 2.0a	31.96 ± 3.0a	6.23 ± 0.1a	2.12 ± 0.3a	1.03 ± 0.05a	0.94 ± 0.09a	2.50 ± 0.21a
Tetraploid	DM22	WW	89.9 ± 2.0a	153.47 ± 3.5c	13.21 ± 0.4b	3.39 ± 0.1b	2.10 ± 0.01c	1.52 ± 0.02c	3.32 ± 0.18a
		FS	84.0 ± 3.0a	115.80 ± 2.7b	10.14 ± 0.6a	2.56 ± 0.1a	1.24 ± 0.04b	1.67 ± 0.06b	3.24 ± 0.19a
		PS	86.8 ± 1.0a	66.79 ± 6.3a	8.27 ± 0.8a	1.80 ± 0.2c	1.03 ± 0.01a	1.81 ± 0.12a	3.06 ± 0.09a
	DM31	WW	77.7 ± 5.0a	76.95 ± 3.6c	7.89 ± 0.5b	4.22 ± 0.3b	2.11 ± 0.03c	1.28 ± 0.04c	3.21 ± 0.03a
		FS	75.4 ± 4.0a	41.87 ± 1.6b	4.79 ± 0.4a	1.88 ± 0.2a	1.33 ± 0.04b	1.54 ± 0.08b	3.17 ± 0.11a
		PS	71.7 ± 3.0a	30.12 ± 2.2a	4.53 ± 0.5a	1.76 ± 0.2a	0.99 ± 0.04a	1.73 ± 0.09a	3.38 ± 0.15a
	ANOVA	Variety	***	***	***	***	***	***	***
		Treatment	ns	***	***	***	***	ns	*
		V × T	ns	***	ns	ns	***	***	ns

Values are mean ± SE (*n* = 3; *n *= 6 for gas exchange characteristics). Means within column having same letter are statistically similar at *P *< 0.05 according to Duncan’s multiple range tests. *, **, *** indicate significant at 0.05, 0.01, and 0.001, respectively

**Fig. 1:**
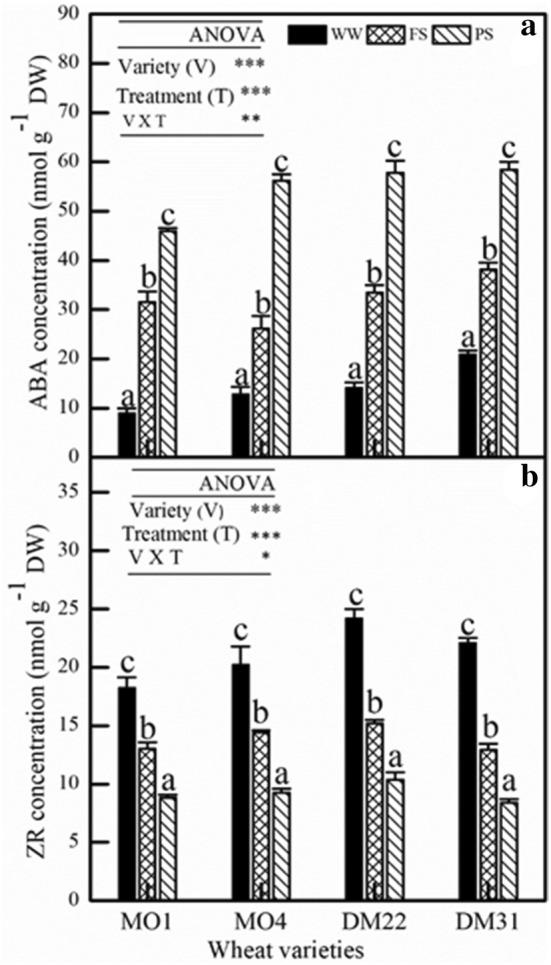
Changes in the concentration of leaf ABA (**a**) and ZR (**b**) at the flowering stage of four primitive wheat varieties, MO1, MO4 (diploid); DM22 and DM31 (tetraploid), respectively. Values are mean ± SE (*n *= 3). Interaction for variety x treatment was significant, *, **, *** indicate significant at 0.05, 0.01, and 0.001, respectively. Different letters indicate significant differences between treatments

The biosynthesis of two major nHRS chemical substances, i.e. ABA and ZR, were investigated when the nHRS operated. A general trend was that leaf ABA biosynthesis was significantly enhanced under nHRS across four wheat varieties in FS and PS (Fig. 1a). This phenomenon was consistent with the definition of existing root-to-shoot communication theory. Leaf ABA concentration was increased up to high level in PS, leading to significant reduction in leaf stomatal conductance. On the other hand, leaf ZR concentration turned to decline substantially across wheat species and was lowest in PS (Fig. 1b). It was decreased by 17%, 17%, 23% and 26% in FS and by 35%, 37%, 40% and 45% in PS of MO1, MO4, DM22 and DM31 respectively, in comparison with that of WW (control) group. These results confirm the antagonistic action of ABA and CK to mediate stomatal closure and drought stress signalling crosstalk.

### Lipid peroxidation (in terms of malondialdehyde), proline, anti-oxidant responses and reactive oxygen species (ROS) production

**Fig. 2:**
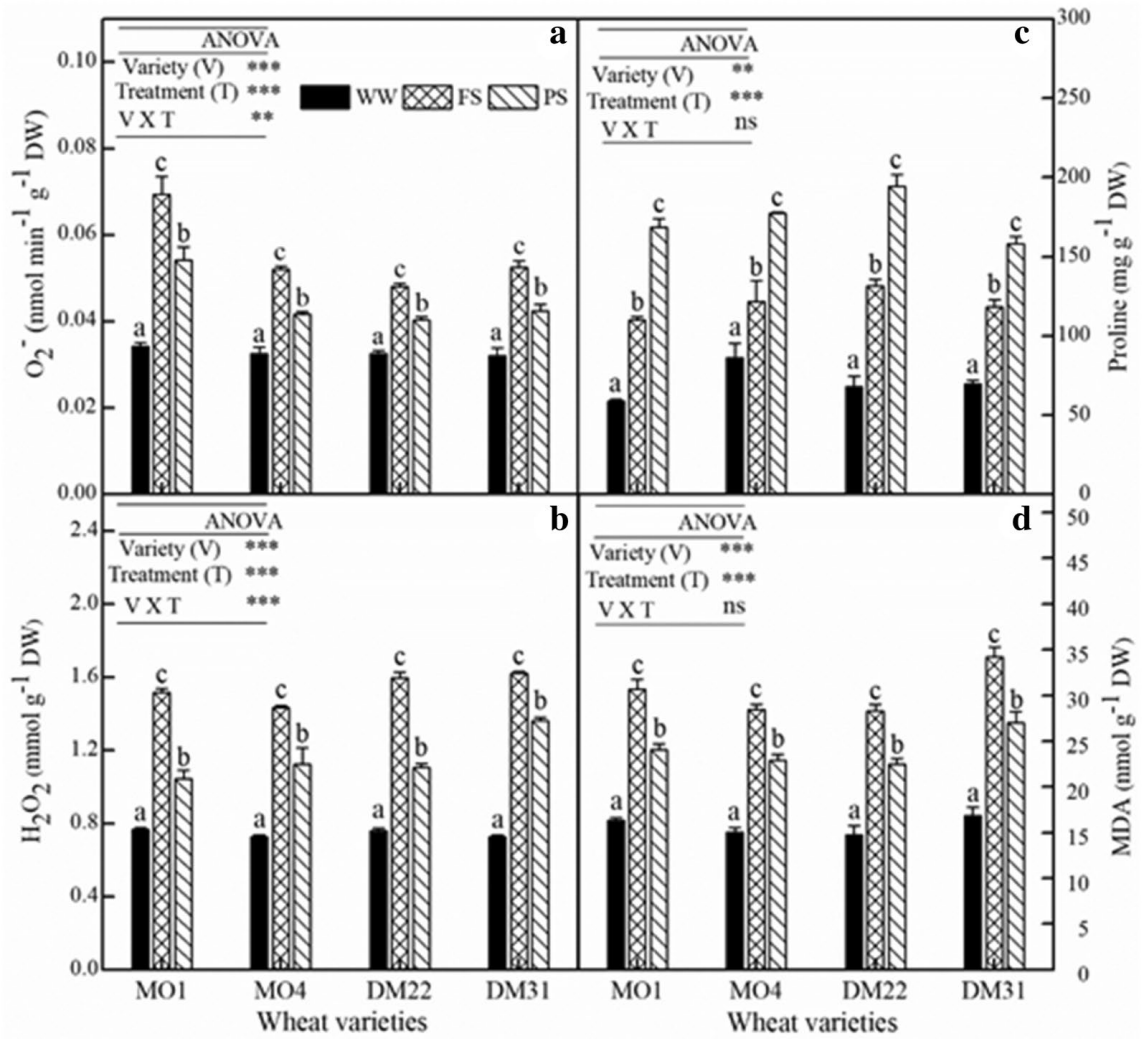
Changes in production rate of reactive oxygen species (O_2_^−^) (**a**), hydrogen peroxide concentration (H_2_O_2_) (**b**), and osmoprotectants, such as proline (**c**) and malondialdehyde (MDA) concentration (**d**) in the leaves at the flowering stage of four primitive wheat varieties, MO1, MO4 (diploid); DM22 and DM31 (tetraploid). Values are mean ± SE (*n* = 3). Trial 1

MDA (Malondialdehyde) is a critical physiological parameter to evaluate the extent of cell membrane lipid peroxidation in plants exposed to drought stress. In general, MDA level was significantly elevated under drought stress across four wheat species. Yet, MDA in PS plants was significantly lower than that in FS plants (Fig. 2d). Moreover, the content of leaf proline was increased in primitive wheat under drought (Fig. 2c). On average, both diploid and tetraploid wheats had relatively similar proline accumulation, but PS treatment resulted in higher proline accumulation than FS one, suggesting that the osmotic regulation ability of PS individuals is greater, hence the PS individuals were under the continuous operation of nHRS.

On the other hand, the O_2_^−^ production was significantly increased in FS and PS in all wheat varieties (Fig. 2a). In addition, drought stress resulted in significant enhancement of leaf H_2_O_2_ production in all four varieties (Fig. 2b). Particularly, PS treatment generally brought about significantly lower H_2_O_2_ concentration and O_2_^−^ production in leaves than FS one did (Fig. 2).

**Fig. 3:**
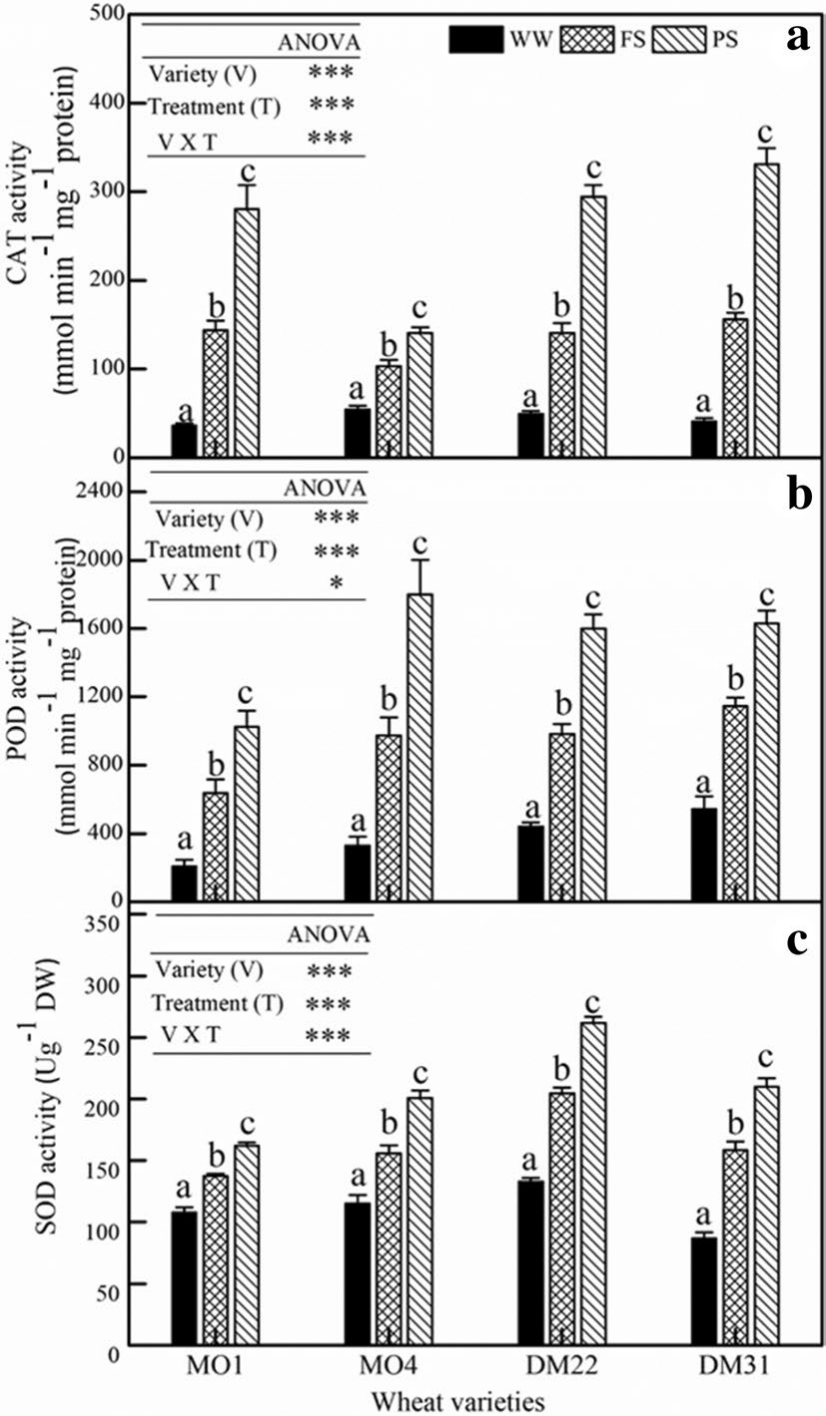
Changes in the activity of the antioxidant enzymes: **a** catalase (CAT), **b** peroxidase (POD), and **c** superoxide dismutase (SOD) in the leaves at the flowering stage of four primitive wheat varieties, MO1, MO4 (diploid); DM22 and DM31 (tetraploid). Values are mean ± SE (*n* = 3), Trial 1

In response to drought stress enhanced activities of antioxidant enzymes such as CAT (catalase), POD (peroxidase), and SOD (superoxide dismutase) were observed (Fig. 3). A general trend was that the anti-oxidant enzyme activities were remarkably greater in PS than FS plants, suggesting that the former had stronger adjustment ability than the latter in response to drying conditions. Overall, nHRS improved the activities of major anti-oxidant enzymes, which indicate the enhancement in an adaptive strategy of these individuals.

### Relationship between ABA, ZR and leaf stomatal sensitivity and PCA analysis

**Fig. 4:**
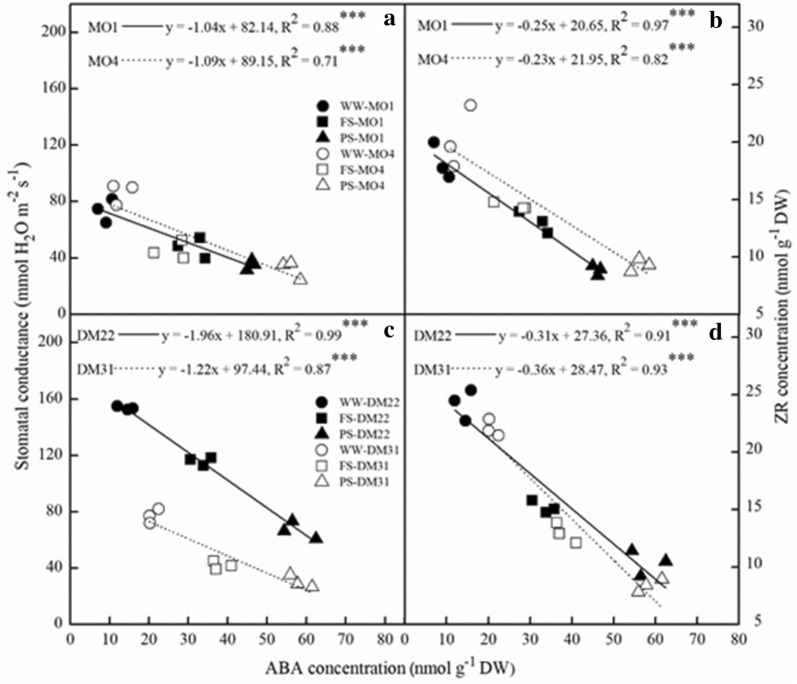
The relationship between leaf ABA concentration and stomatal conductance (**a**, **c**), and between leaf ABA and ZR concentration (**b**, **d**) in four primitive wheat varieties, MO1, MO4, DM22 and DM31 subjected to three water stress treatments. The fitted linear regressions are given: *P < 0.05; **P < 0.01; ***P < 0.001. Trial 1

We conducted correlation analyses on the relationships between leaf ABA and ZR, and between leaf ABA and stomatal sensitivity. There existed a significantly negative correlation between leaf ABA and ZR biosynthesis of all plants. The linear regression coefficient reached up to significant level in MO1, MO4, DM22 and DM31, respectively. As expected, leaf ABA concentration was significantly negatively correlated with stomatal conductance across four wheat varieties. The regression coefficient was 0.88 in MO1, 0.71 in MO4, 0.99 in DM22 and 0.87 in DM31, respectively (Fig. 4).

**Fig. 5:**
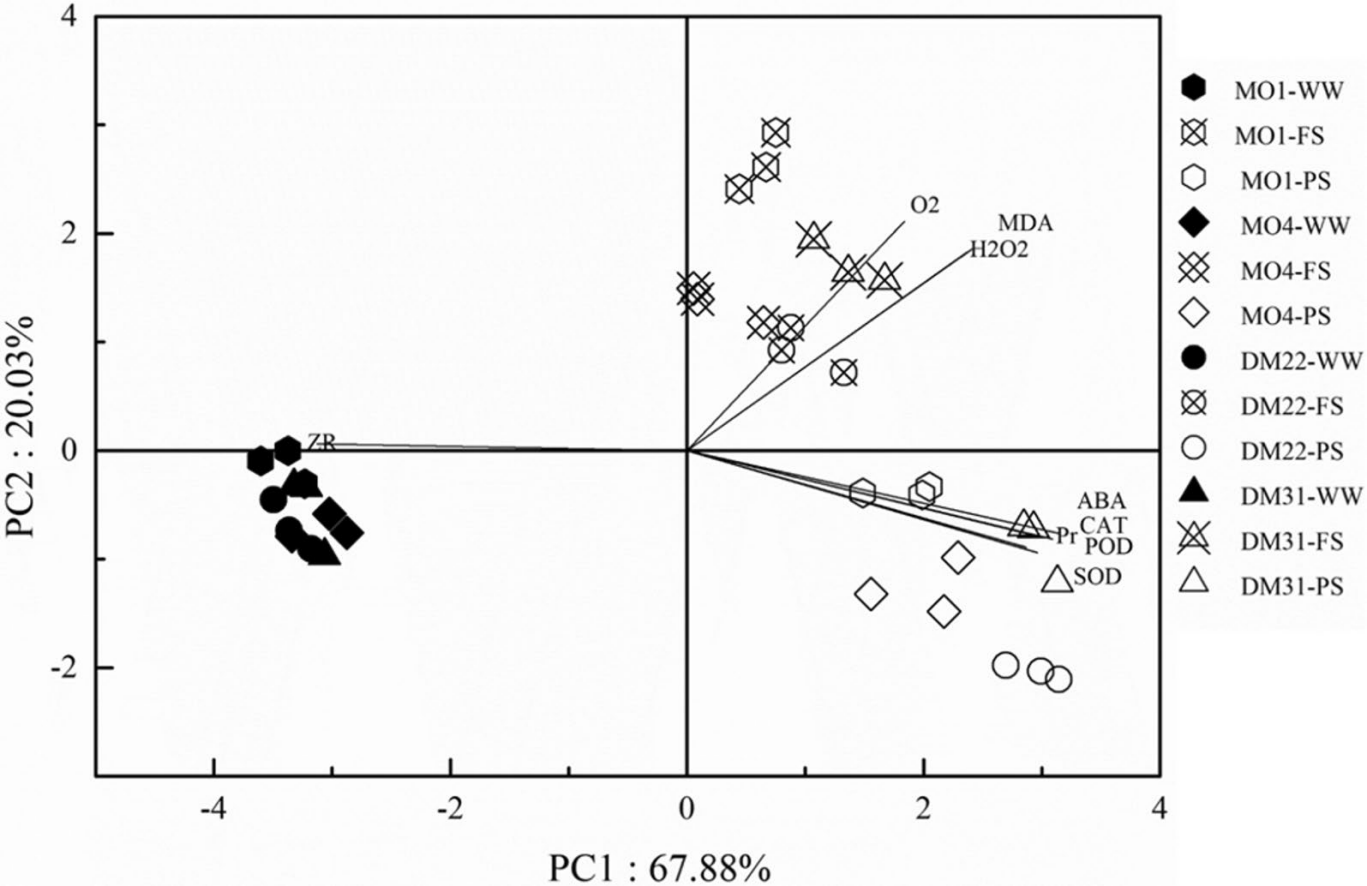
PCA analysis on biochemical parameters among three drought treatments at the flowering stage of four primitive wheat varieties, MO1, MO4, DM22 and DM31 Trial 1. ZR, Cytokinin; ABA, Abscisic acid; SOD, superoxide dismutase; CAT, catalase; POD, peroxidase; H_2_O_2_, hydrogen peroxide; O_2_^−^, oxidase; Pr, proline; and MDA, malondialdehyde

PCA Analysis on biochemical parameters of primitive wheat confirms a complex network of interconnected signalling pathways, in which abscisic acid and cytokinin played a key role as an nHRS materials (Fig. 5). Moreover, the WW treatment of all wheat varieties lay in the same area of scale, whereby two drying methods FS and PS were placed opposite to WW and had clear differences between two methods. ZR showed a significant negative relation with other biochemical responsive molecules, whereas ABA had a strong positive interactions with proline and antioxidants enzymes, respectively.

### Plant growth, grain yield and water use in response to partial and full root-zone drought stress methods

In order to compare the physiological and agronomic performance during the activation of nHRS in FS and PS, we determined plant growth, grain filling and water consumption at maturity stage. Total water consumption was recorded and analyzed in all the treatment groups/wheat species across whole growing period. Root-zone water treatments led to substantial reduction in total water consumption amount. For example in MO1, total water consumption was 1.83 L per plant in control group, whereas it was reduced to 1.34 and 1.12 L per plant in FS and PS, respectively (Table 1). A pronounced tendency was that water consumption was significantly greater in FS than PS which describes that full root-zone drought stress generally led to greater water consumption than alternative partial root-zone stress (Table 1).

In control group, grain yield per plant was 2.08, 1.95, 3.20 and 2.69 g in MO1, MO4, DM22 and DM31, respectively. In contrast, it was lowered to 1.39, 1.13, 2.08 and 2.04 g in MO1, MO4, DM22 and DM31 respectively, in FS group whereas it was markedly reduced in PS group (1.11, 0.97, 1.87 and 1.72 g, respectively) in the corresponding four varieties. Furthermore, the changes in above-ground biomass were similar as those of grain yield (Table 2). Finally, root biomass was totally reduced in both FS and PS while no significant difference was observed across all four varieties (Table 2).

**Table 2:** Effects of water stress on yield and yield components of primitive wheat (diploid and tetraploid) cultivars

Species	Varieties	Treatments	Grain yield (g/plant)	Above-ground biomass (g/plant)	Root biomass (g/plant)	Harvest index	Root-to-shoot ratio
Diploid	MO1	WW	2.08 ± 0.02c	5.49 ± 0.03c	0.43 ± 0.03b	0.38 ± 0.01a	0.08 ± 0.01a
		FS	1.39 ± 0.05b	3.41 ± 0.06b	0.29 ± 0.03a	0.39 ± 0.02a	0.08 ± 0.01a
		PS	1.11 ± 0.02a	3.04 ± 0.07a	0.21 ± 0.04a	0.37 ± 0.01a	0.07 ± 0.01a
	MO4	WW	1.95 ± 0.03c	5.14 ± 0.09c	0.40 ± 0.03b	0.38 ± 0.01a	0.08 ± 0.00a
		FS	1.13 ± 0.05b	2.93 ± 0.03b	0.27 ± 0.04a	0.38 ± 0.02a	0.09 ± 0.01a
		PS	0.97 ± 0.05a	2.57 ± 0.10a	0.23 ± 0.03a	0.37 ± 0.01a	0.07 ± 0.01a
Tetraploid	DM22	WW	3.20 ± 0.04c	6.98 ± 0.38c	0.46 ± 0.02b	0.47 ± 0.02a	0.07 ± 0.01a
		FS	2.08 ± 0.04b	4.02 ± 0.11b	0.35 ± 0.03a	0.59 ± 0.01b	0.09 ± 0.01a
		PS	1.87 ± 0.11a	3.16 ± 0.11a	0.28 ± 0.03a	0.62 ± 0.05b	0.09 ± 0.01a
	DM31	WW	2.69 ± 0.06c	6.78 ± 0.16c	0.48 ± 0.06b	0.42 ± 0.02a	0.07 ± 0.01a
		FS	2.04 ± 0.06b	4.20 ± 0.04b	0.31 ± 0.05a	0.52 ± 0.01b	0.07 ± 0.01a
		PS	1.72 ± 0.02a	3.36 ± 0.02a	0.25 ± 0.04a	0.53 ± 0.01b	0.07 ± 0.01a
	ANOVA	Variety	***	***	*	***	ns
		Treatment	***	***	***	***	ns
		V × T	*	ns	ns	**	ns

Values are mean ± SE (*n *= 15). Means within column having same letter are statistically similar at *P* < 0.05 according to Duncan’s multiple range tests. *, **, *** indicate significant at 0.05, 0.01, and 0.001, respectively

In contrast, water use efficiency (WUE) varied in wheat species and water treatments. The WUE_G_ has been mostly used in assessing the level of water use in higher plant in previous studies, and it was viewed as a typical parameter of water use. The data indicated that the WUE_G_ under nHRS was totally lowered in two diploid wheat varieties, while it was significantly increased in two tetraploid ones and was greater in PS than FS (Table 1). WUE_AGB_ was similar as that of WUE_G_ in two diploid varieties while tetraploid varieties had no significant difference across all treatments (Table 1).

### Biomass allocation and reproductive output of two primitive wheat species in response to nHRS

There was no significant difference in root-to-shoot ratio among WW, FS and PS group across both wheat species. Critically, no significant difference was observed in harvest index (HI) among WW, FS and PS in two diploid wheat varieties, suggesting that integral energy allocation pattern was not changed while total energy sequestration (i.e. photosynthetic product, particularly for above-ground biomass) was lowered under drought stress (i.e. in FS and PS). Tetraploid wheats displayed greater HI in two drought stress methods than sufficient water supply. It was noted that HI was increased in PS than FS in DM22 and DM31, respectively (Table 2).

**Fig. 6:**
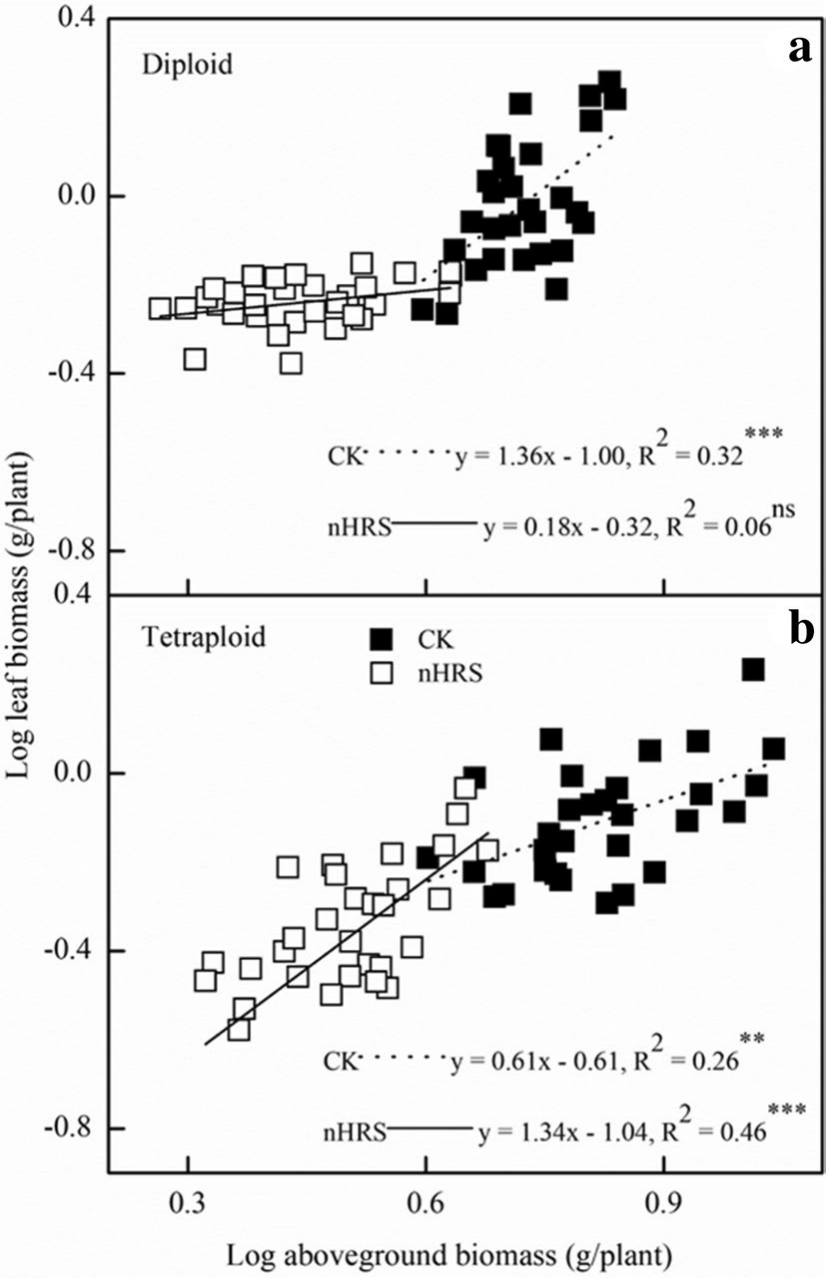
The allometric relationship between leaf biomass and aboveground biomass in two primitive wheat species diploid (**a**) and tetraploid (**b**) under well-watered (CK) and drought stress (nHRS) treatments. The symbols represent the two water treatments (open rectangular panel, well-watered (CK); closed rectangular panel, drought stress (nHRS)). The data harvested came from maturity stage. The fitted linear regressions are given: *P < 0.05; **P < 0.01; ***P < 0.001. Trial 2

**Fig. 7:**
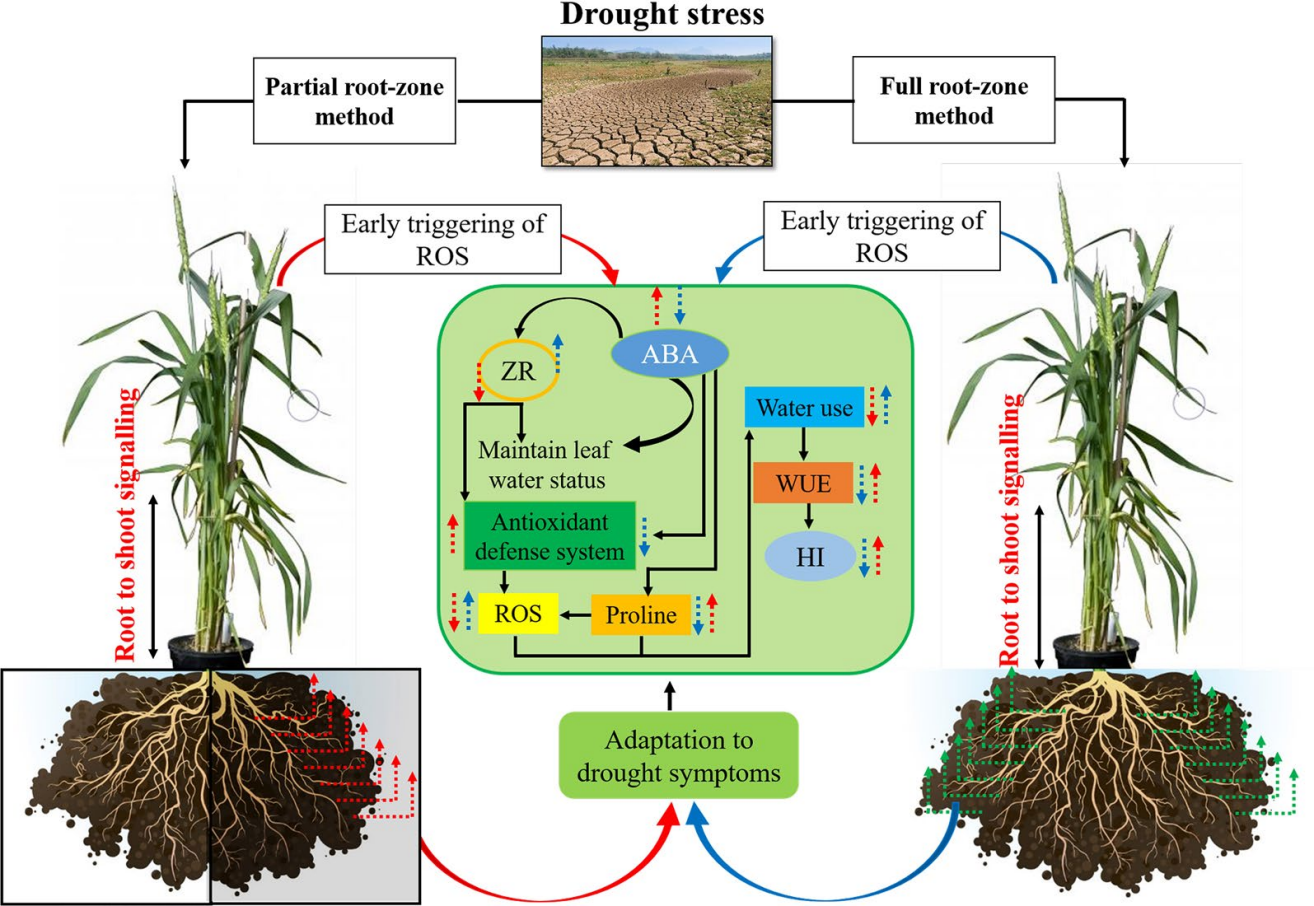
Schematic diagram showing the mechanisms of plant adaptive responses to partial (PS) and full (FS) root zone drought stress methods used in this study. Red and blue solid arrow lines indicate PS and FS individuals’ responses respectively. Red and blue dotted arrow lines with upward and downward arrow directions indicate the higher and lower responses of respective compounds between the two drought stress methods. *ROS* reactive oxygen species; *ABA* abscisic acid; *ZR* cytokinin; *WUE* water use efficiency; *HI* harvest index

In order to evaluate the biomass allocation pattern under the nHR operation, we also analyzed the allometric relationship between individual size and metabolic rate (generally expressed by leaf biomass) in this study. The allometric relationships between leaf and aboveground biomass were conventionally presented by the exponent α. We found significant differences in the value of α between two wheat species and between sufficient water supply and drought stress across wheat species. Interspecific differences in allometric exponents acted as a critical parameter to explain the biomass allocation pattern and adaptive strategy (Fig. 6). Firstly, diploid and tetraploid species displayed significant differences in the allometric exponent α. Under sufficient water supply, the α value of diploid was 1.36, significantly > 1.0 (constant value), indicating that more energy was transformed into to leaf tissues. As is well known, the value 1.0 implied an isometric relationship between two variables. In tetraploid species, the α value was only 0.61, massively lower than 1.0. The result implied that less biomass was allocated into leaf tissues under sufficient water supply condition. Importantly, nHRS regulation substantially altered α value across wheat species. The value of α turned up to 1.34 in tetraploid wheats, while it was lowered to 0.18 in diploid. Therefore, tetraploid species allocated more energy to leaves than diploid species under the regulation of nHRS (Fig. 6). The intricate mechanisms of root to shoot signalling under the partial and full root-zone drying methods is explained by schematic diagram (Fig. 7).

## Discussion

Plants are very dynamic systems having a great ability to cope with drying conditions by triggering a network of interconnected signalling pathways, in which ABA play a key role and function as a principal mediator of these responses. ABA induce the stomatal closure which ultimately reduce water loss [1] and maintain plant water relation [35, 36]. In our study we aimed to uncover the role of ABA in long distance signalling under two contrasting drying methods of partial and full root-zone in primitive wheat species. Partial root-zone drying method appeared more likely to simulate the occurrence of nHRS and the induction of soil drying than full root-zone drying method. A great enhancement in leaf ABA synthesis was reported in partial root-zone drought method which illustrates that this method activates early triggering of nHRS. ABA-mediated stomatal closure involves activation and cross talk of interconnected signalling pathways [37]. Increased ABA accumulation triggers the cascade events and closes the stomata and this mechanism has been widely supported by studies at physiological, biochemical and molecular level since ABA was found [38].

Over last decades, split-root system displayed a particular importance in the field of root–shoot communication theory [20]. Our study showed that partial root-zone system in dry soil can trigger stomatal closure while leaf water status did not decline significantly. This is also confirmed in earlier studies that stomatal closure responses were substantially strong when half of root system was subjected to drying soil [39]. During this process, cytokinin was also involved in the regulation of stomatal behavior, with ABA acting in conjunction [40, 41]. In view of signal crosstalk theory, both ABA and ZR synergistically triggered stomatal closure (Fig. 4) and mediate drought tolerance [41]. Abscisic acid acted as a modulators of the coordinated adaptation under water shortage. Cytokinin also acted as a soil drying detection signal establishing antagonistic crosstalk with ABA. Enhanced cytokinin concentration in xylem can reduce stomatal sensitivity against ABA and increase stomatal opening directly [42]. Actually, both ABA and CKs can regulate plant adaptive responses to water deficit [43]. In present study, leaf ABA accumulation was increased, while ZR was lowered significantly in PS method across all wheat varieties in response to nHRS activation which resulted into stomatal closure and reduced water use (Table 1).

Considering the strong negative relation of ABA concentration and stomatal conductance (Fig. 4a, c), it can be argued that nHRS enhanced stomatal sensitivity to ABA. This is consistent with a notion of high stomatal sensitivity in response to ABA accumulation in hexaploid wheat varieties [44], yet they did not conduct such an investigation in primitive wheat species. Drought stress induces ROS production including hydrogen peroxide, hydroxyl radicals and superoxide [27], leading to oxidative stress towards plant tissues and cells. The ROS, especially H_2_O_2_, would act as a signal to partially close the stomata in guard cells [45]. As is well known, ABA signalling pathways consist of many components such as reactive oxygen species, nitric oxide and antioxidants [46]. Thus, a signalling crosstalk of different biochemical compounds might offer a driving force for root-to-shoot communication under drying soil. Physiological mechanism of plant response to drought stress is complex, which involves adaptive strategies and deleterious reactions. In current study, ROS generation was markedly enhanced under the regulation of nHRS across four wheat varieties in PS and FS (Fig. 2a, b). This phenomenon was similar as observed in hexaploid spring wheat varieties [47] and winter wheat varieties [48]. Moreover, accumulation of ROS was lower in PS than FS (Fig. 2a, b).

Finally, the activities of antioxidant enzymes including POD, CAT and SOD were truly enhanced, which was in concert with the production of ROS (Fig. 3). Therefore, there might exist a synergistic effect between the two types of stress signals. Previous study showed that POD and CAT were responsible for the removal of H_2_O_2_, whereas SOD catalyses the O_2_^−^ to H_2_O_2_ dismutation [27]. The proline content was much higher in PS than FS (Fig. 2c) which might be associated with the increased ABA content in PS and help improved the drought tolerance in plants. These finding were similar with the existing observations by [49, 50]. In both PS and FS, there existed significant differences in the levels of CAT, POD and SOD among wheat varieties. In this case, the interaction between the nHRS and the antioxidant enzymes might perform a key role to mitigate the ROS effects in all drought-stressed plants (Figs. 2a, b, 3). Alternatively, enhanced antioxidant defense and ABA accumulation reduced the level of ROS in the leaves in continuous operation of nHRS (PS) and this signalling crosstalk might reduce the lipid damage, as expressed by MDA concentration in two drought treatments (Fig. 2a, b, d). Our results were similar with some other studies conducted in the droughted wheat population [44, 47, 48]. Recently, many researchers have shown the changes in the antioxidant enzyme activities and expression of stress-related genes under drought stress [51]. Our results provide evidence that the enhancement of antioxidant enzyme activities would have been related with up-regulation of antioxidant defence genes in primitive wheat.

Photosynthetic product transfer and re-allocation accounted for energy distribution pattern, which was viewed as a typical drought-adaptive strategy in dryland crops. In two drought stress methods (FS and PS), in response to nHRS activation, water consumption reduced in all wheat varieties but WUE_G_ and HI was only improved in tetraploid species (Tables 1, 2) suggesting that less energy was allocated in seed production in diploid primitive wheat. This implies that primitive diploid wheats had weaker adaption ability to drought stress than tetraploid ones. Moreover, nHRS regulation significantly decreased grain yield, root biomass and above-ground biomass (*P* < 0.05), except for root-to-shoot ratio across wheat varieties (Table 2). Allometric relationship between leaf biomass and above-ground biomass indicated that more energy was transformed into the leaves in tetraploid varieties than diploid ones under drought (Fig. 6). These results showed that less biomass was allocated to leaves during the domestication process from diploid to tetraploid (Fig. 6). Additionally, leaf gas exchange characteristics, stomatal conductance, photosynthetic rate, and transpiration rate, were reduced (Table 1) while leaf ABA concentration was increased (Fig. 1a) under nHRS regulation which improved the desiccation tolerance in the form of osmotic adjustment (Fig. 2) and antioxidant defence abilities (Fig. 3) in plant leaves. These results were consistent with the observation by some other researchers [3, 22, 52]. Grain yield was substantially reduced in diploid wheat, briefly due to longer growth period of plants and more decomposition and compartmentalization of ABA in late growth period [53]. Moreover, PS treatment had less total water consumption than FS treatment did regardless of wheat species. In fact, partial root-zone drought stress method for soil drying helped plant to better develop the drought adaptive mechanisms in terms of biochemical and physiological responses to alleviate stress symptoms as compare to full root-zone drought stress. Finally, the differences at eco-physiological and agronomic levels in both primitive wheat species were quite significant.

## Conclusion

Plant intracellular and extracellular signalling crosstalk intervene the trade-off between crop growth and drought tolerance through the life period of a plant. A clear understanding of the intricate mechanisms of these trade-offs will help to set up the novel crop varieties to optimize yield production. In present study, we used two drying methods, partial and full root-zone, to induce soil drying in plant container-culture experiments. Partial root-zone stress (PS) was performed using split-root alternative water supply method (one half wetting and the other drying) to induce the continuous operation of nHRS, and full root-zone stress (FS) was exposed to whole soil block to induce periodic operation of nHRS since jointing stage of primitive wheat plants. Two methods lead to different observational results in plant water relation, such as non-hydraulic root-sourced signal (nHRS) and PS was more likely to simulate the occurrence of nHRS and the induction of soil drying than FS. During PS, higher ABA concentration and lowest stomatal conductance were found, thereby reducing the water use. These findings lead us to conclude that partial root-zone stress method to induce nHRS is a more feasible method with a great capacity to reduce water consumption and enhance adaptation to a constantly changing environment for global crop production systems. These observations also suggest that different root-zone planting methods can be considered to improve the plant phenotypic plasticity and tolerance in water-limited rainfed environments.

## Methods

### Plant materials and growth conditions

Two relatively independent but closely related pot-culture trials were conducted from March to August 2013 at the Yuzhong Experimental Station of Lanzhou University, Yuzhong County, Gansu Province (35°51′N, 104°07′ E, altitude 1620 m), northwest China. Four wheat varieties included two diploid (*Triticum monococcum* L.) MO1 and MO4, and two tetraploid (*Triticum dicoccum* Schrank ex Schübl.) DM22 and DM31 were used in this study. Seeds resources of diploid and tetraploid wheats were provided by the Institute of Crop Germplasm Resources, Chinese Academy of Agricultural Sciences, Beijing, China. The varieties were grown in a rainout shelter (50 m long × 24 cm wide × 5.7 m high) that can be opened and closed according to weather event.

Seeds were prepared and vernalized at 4 °C for 24 h and kept on moistened filter paper by distilled water under dark for germination in an incubation cabinet at 25 °C. Eighteen seeds per pot were sown in 72 plastic pots (28 cm diameter × 30 cm high) containing 11 kg of sieved loess soil-based substrate (loess soil:vermiculite (v/v) = 4:1). Soil water content (SWC) at field was determined by watering to the excess and then allowed the pots to drain until 2 days before weighing. After germination, the seedlings were thinned to maintain 12 plants per pot for both trials. Before planting the seeds, 1.25 g N, 0.36 g P and 0.44 g K per pot was applied to avoid the nutrition deficiency. After seeds emergence, all the plants were daily watered to maintain soil water content within 80% field capacity (FC) before drought stress initiation. Harvest was taken at the flowering stage of each cultivar according to their developmental period since imposing water stress at jointing stage. To measure different biochemical and physiological attributes, fully expended leaves were collected from three pots per treatment and wheat variety and immediately frozen in liquid nitrogen. Leaf RWC, and gas exchange parameters were measured around 11:00 a.m.

### Trial 1

Split-root trial was conducted to evaluate the physiological characteristics of major root-sourced chemical signals and its association with other drought-stressed signals in primitive wheat species. Water treatments were exposed at jointing stage, including (1) control group with 80% FC maintenance throughout growing period (WW group); (2) split-root treatment group, i.e. partial root-zone drought stress (PS group) with alternative one half wetting and another drying in two parts of root system; and (3) full root-zone drying group (FS group) with 55% FC to induce nHRS. Split-root treatment was carried out as watering half root system (65–45% FC) and remaining another half drying alternatively. A divider was placed in the middle of each pot, to ensure no substance exchange between two parts. Soil media was equally filled in both halves of each pot and seeds were sown at the boundary above the divider (Additional file 1: Figure S1). In this group, each pot was allowed to dry until around 45% FC in one of the halves, while another one was rewatered to 65% FC. Following 2 days of treatment (according to preliminary observations), two halves were kept drying and wetting alternatively. In drought stress treatment of whole root system (FS), soil moisture of each pot was maintained at almost 55% FC, with soil water content (SWC) fluctuating from 65 to 45%. Across all treatments, root samples were taken and washed for biomass determination. In split-root treatment, root samples were separately taken and determined in each half of pot. SWC was measured gravimetrically by weighing the pots and expressed as a percentage of available water with FC. Water treatments were exposed since jointing stage according to developmental stage of each variety. For each wheat variety, considering their respective developmental stage, 12 pots were used to take the samples with three replicates.

After imposing water treatments, SWC was measured daily while leaf relative water content (RWC) and gas exchange characteristics were also recorded for each pot, including stomatal conductance (*gs*), transpiration rate (*E*), and photosynthetic rate (*Pn*). Each measurement had three and six replicates respectively, by selecting the upper fully expended leaf (the 2nd leaf from the top for LRW while 1st leaf from the top for gas exchanges characteristics. Stomatal conductance for each replicate was the mean of five readings, and each leaf was measured between 9:00 and 10:00 a.m. using the LI-6400 portable photosynthesis system (Li-Cor, Lincoln, NE, USA) [54]. Two-thirds of leaf discs (5 mm in diameter) were used to measure fresh weight (FW) by sampling and weighing immediately, and then the discs were kept in the tubes having fresh distilled water for 8 h under 10 μmol m^−2^ s^−1^ PFD. Leaf samples were instantly dried using filter paper, and then weighed as the SW (saturated weight). Subsequently, the samples were dried for 24 h at 80 in a forced-draught oven. Leaf RWC was calculated as RWC = ((FW − DW)/(SW − DW)) × 100, where SW was saturated weight, DW was dry weight and FW was the fresh weight respectively [24].

### Trial 2

In order to determine the effects of ABA and other nHRS on yield and yield components in four different primitive wheat varieties, three water gradients were designed from jointing to maturity stage. Water deficit was imposed by withholding water supply until soil water content (SWC) reached up to the predetermined level: (1) 12 pots were maintained about 80% FC by sufficiently watering daily in the evening before sunset; (2) 12 pots were used for split-root trial by imposing the divider. Each pot was allowed to dry until the SWC reached up to 45% FC in the half part of pot and watered in another part until 65% SWC. The cyclic operation was maintained and let pots to dry again until 45% FC and rewatered from another side until 65% FC; and (3) 12 pots were used for fully wetting and drying in whole root system, without the divider used in pots. Each pot was watered till 65% FC and allowed to gradually dry till 45% FC. After that, the same operation was implemented periodically. Each treatment per cultivar was conducted with three replications till maturity stage.

At physiological maturity (~ 110 DAS), whole plants were harvested as defined that completely disappearance of glumes green color. Plant roots were washed free of soil by using screen (0.4 mm). Yield and yield components per plant were recorded, and then divided into shoots (included leaves and husks), grain and roots, after dried for 2 days at 80 °C and then weighed. Data for water use were collected by recording the daily water added during the whole plants life. Following variables were determined: (i) HI (harvest Index) = grain weight/aboveground weight, (ii) root to shoot ratio = root weight/shoot weight and (iii) WUE_G_ (water use efficiency for grain) = grain weight/total water used since sowing until harvest and (iv) WUE_AGB_ (water use efficiency for aboveground biomass) = aboveground biomass/total water used from sowing to harvest.

Allometric relationships between leaf and above-ground biomass were calculated and analysed after transforming the data into log for homogenize variances. To determine the slope (scaling exponents) and intercept (allometric constants) linear regression was used. According to allometric equation [55]:

1 $${\text{Y}} =\upbeta{\text{X}}^{\upalpha}$$

It is generally calculated as:

2 $$\log {\text{y}} = \log\upbeta +\upalpha\log\upchi,$$

In the equation, χ and y are generally referred to as two traits, whereas β is often considered as allometric coefficient, log β as the ‘intercept’ and α as the ‘allometric exponent in Eq. (1) or the ‘slope’ in Eq. (2). Scaling exponent significantly different from 1 (one) states an allometric relationship between two traits.

### Measurement and methods

#### Determination of reactive oxygen species and enzyme assays

Production of O_2_^−^ was measured following the method [56] by observing nitrite formation from hydroxylamine in O_2_^−^ presence. Data for H_2_O_2_ was measured by observing the titanium-peroxide complex absorbance at 415 nm [57]. By using the standard curve of known H_2_O_2_ concentrations, absorbance values were calibrated.

Frozen leaf material (0.5 g) was crushed to make fine powder by using mortar and pestle with liquid nitrogen. Soluble proteins extraction was done by homogenizing with 10 mL of 50 mM potassium phosphate buffer (pH 7.0) having 1% polyvinylpyrrolidone (PVP) and 1 mM EDTA, and addition of 1 mM ascorbate acid to perform APX assay. This homogenous mixture was centrifuged at 4 °C and 12,000*g* for 1200 s and the supernatant was used for following antioxidant enzyme essays. Catalase (CAT, EC 1.11.1.6) activity determined by H_2_O_2_ disappearance (extinction coefficient 39.4 mM^−1^ cm^−1^) for 180 s at 240 nm [58]. Total superoxide dismutase (SOD, EC 1.15.1.1) activity was measured by observing its ability to stop the photochemical reduction of nitro blue tetrazolium (NBT) [59]. Peroxidase (POD, EC 1.11.1.7) activity was determined by following early described method [60].

#### Determinations of lipid peroxidation and free proline

Level of lipid peroxidation in leaves was determined by measurement of malondialdehyde (MDA) amount [61]. MDA content was calculated by its absorbance and mentioned as nmol MDA g^−1^ DW [61]. Free proline was determined by following the earlier described method [62].

#### ABA and ZR extraction, purification and quantification

ABA and ZR extraction and purification methods were modified and followed from those already described [63]. Leaf segments were ground using silica in liquid nitrogen with a mortar and pestle, then extracted with ice-cold 80% methanol (v/v) contained 1 mM butylated hydroxytoluene for avoiding oxidation, subsequently moved to a centrifuge tube and kept overnight at 4 °C. The extract solution was then centrifuged for 900 s at 4 °C and 10,000*g*, then supernatant removed by a pipette into a centrifuge tube. To centrifuge again at 4 °C and 10,000*g* for 900 s, the remnant was further suspended for 1 h at 4 °C into the same ice-cold extracting solution, then supernatant removed by a pipette in the same centrifuge tube. This combined supernatant was then passed from Chromosep C18 columns (C18 Sep-Park Cartridge, Waters, Milford, MA, USA), prewashed using 5 mL 80% and 10 mL 100% methanol. The collected efflux was dried by evaporation using nitrogen. To determine the level of ABA and ZR, residues were dissolved in 10 mM phosphate-buffered saline (pH 7.5) contained 0.1% (w/v) gelatin and 0.1% (v/v) Tween 20. ABA and ZR measurement was done immunologically by using ELISA (enzyme-linked immunosorbent assay) technique. ABA quantification by ELISA technique has already been described [64]. ZR quantification is followed by the method of ABA though antigens were used according to their respective antibodies. In current study, ABA and ZR percentage recovery was monitored and calculated by addition of known standard ABA and ZR quantities to a split extract. Monoclonal antibody specificity was confirmed and other nonspecific inhibitors possibility was excluded in earlier studies [64].

#### Statistical analyses

Presentation of data was the means of three replicate samples in trial 1 while 15 replicate samples (3 pots × 5 plants for each pot) was analyzed in trial 2. All the data were examined by two-way ANOVA (analysis of variance) (water treatments and wheat varieties). SPSS (SPSS 22.0 version, Chicago, IL) for Window was used to conduct all the data analyses and the means were compared by Duncan’s multiple range tests at *P* = 0.05. Origin 8.5 (Microcal Software Inc) was used to draw the figures and performed the correlation analysis. To study the allometric relationship, the standardized major axis tests and routines (SMATR) software package was used.

## Additional file

**Additional file 1.** Schematic diagram of the specially designed split pots used to expose two halves of the root system for partial root-zone (PS) drought stress method.

## Data Availability

The data generated or analyzed during this study are included in this published article and its supplementary information files.

## Abbreviations

ABA: abscisic aid
CAT: catalase
CK: cytokinin
FS: full root-zone stress
LRWC: leaf relative water content
nHRS: non-hydraulic root signals
PS: partial root-zone stress
POD: peroxidase
ROS: reactive oxygen species
SOD: superoxide dismutase
ZR: zeatin
