# Supplementary material for: Partial and full root-zone drought stresses account for differentiate root-sourced signal and yield formation in primitive wheat
Source: Plant Methods. 2019 Jul 12;15:75. doi: 10.1186/s13007-019-0461-5 (PMC6624928; doi:10.1186/s13007-019-0461-5)
Supplement: Supplementary file 1 — Additional file 1. Schematic diagram of the specially designed split pots used to expose two halves of the root system for partial root-zone (PS) drought stress method. [file 13007_2019_461_MOESM1_ESM.docx]

**Partial and full root-zone drought stresses account for differentiate root-sourced signal and yield formation in primitive wheat**

Asfa Batool^1^, Zheng-Guo Cheng^1^, Nudrat Aisha Akram^2^, Guang-Chao Lv^1^, Jun-Lan Xiong^1^, Ying Zhu^1^, Muhammad Ashraf^1,3^ and You-Cai Xiong^1^*

^1^State Key Laboratory of Grassland Agro-Ecosystems, School of Life Sciences, Lanzhou University, Lanzhou 730000, China; ^2^Department of Botany, GC University, Faisalabad 38040, Pakistan; ^3^Faculty of Agriculture, the University of Sargodha, Sargodha 40100, Pakistan.

* Correspondence: [xiongyc@lzu.edu.cn](mailto:xiongyc@lzu.edu.cn)

Fax / Phone: +86-931-8914500

**Figure legends**

**Additional file 1: Figure S1** Schematic diagram of the specially designed split pots used to expose two halves of the root system for partial root-zone (PS) drought stress method


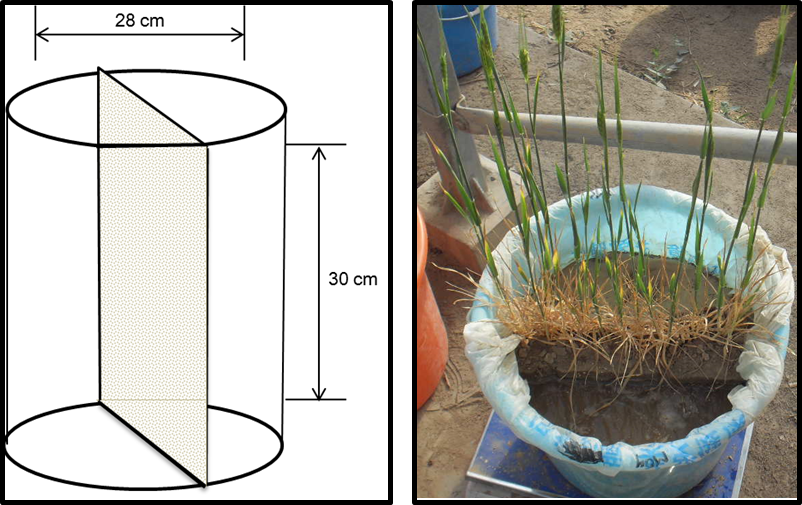


**Additional file 1: Figure S1** Schematic diagram of the specially designed split pots used to expose two halves of the root system for partial root-zone (PS) drought stress method
